# The Development of Aromas in Ruminant Meat

**DOI:** 10.3390/molecules18066748

**Published:** 2013-07-04

**Authors:** Virginia C. Resconi, Ana Escudero, María M. Campo

**Affiliations:** 1 Departamento de Producción Animal y Ciencia de los Alimentos, Facultad de Veterinaria, Universidad de Zaragoza, c/ Miguel Servet 177, 50013-Zaragoza, Spain; E-Mail: marimar@unizar.es; 2 Departamento de Química Analítica, Facultad de Ciencias, Universidad de Zaragoza, c/ Pedro Cerbuna 12, 50009-Zaragoza, Spain; E-Mail: escudero@unizar.es

**Keywords:** lipid oxidation, Maillard reaction, ruminant meat flavour, Strecker reaction, thiamine degradation

## Abstract

This review provides an update on our understanding of the chemical reactions (lipid oxidation, Strecker and Maillard reactions, thiamine degradation) and a discussion of the principal aroma compounds derived from those reaction or other sources in cooked meat, mainly focused on ruminant species. This knowledge is essential in order to understand, control, and improve the quality of food products. More studies are necessary to fully understand the role of each compound in the overall cooked meat flavour and their possible effect in consumer acceptability.

## 1. Introduction

The aromas that are released when meat is cooked, and the appearance and the aromas of the meal on the plate influence the acceptability. Once the meat enters the mouth, texture (tenderness, juiciness, fibrousness, greasiness, *etc*.), aroma, and taste are the main factors that influence the sensory quality of the product.

Aroma is the most important contributor for the identification of the animal species, followed by texture; whereas tastes from beef, pork, lamb and chicken are almost indistinguishable [1]. Goat, for example is characterized by a strong gamy aroma, as other wild animals; whereas this attribute is very weak or even absent in chicken, pork, rabbit, turkey, veal and lamb meat [2]. Within species, aroma or flavour (aroma + taste) can be further discriminated according feeding and age of the animal, and those differences can influence consumer acceptability [2,3,4,5]. Therefore, is highly relevant to understand the origin of the aroma compounds.

Some odour-active compounds are present in the raw meat, such as 4-ethyloctanoic acid (mutton-smell) in sheep and are not much affected by cooking [6], however it is generally accepted that the aroma of meat is mainly developed upon heating treatment, where thiamine (vitamin B1), glycogen, glycoproteins, nucleotides, nucleosides, free sugars/phosphate, amino acids, peptides, amines, organic acids and lipids are the precursors. During the *post-mortem* period, the concentrations of precursors change, primarily, because of hydrolytic activity [7]. When meat is heated, those precursors participate in reactions that form intermediates, which can continue to react with other degradation products to form a complex mixture of volatiles, including those that are responsible for the aroma of meat [7].

The primary reactions involved in the formation of aroma compounds in cooked meat are the oxidation of lipids, the degradation of thiamine, the Strecker reaction and the Maillard reaction. Those reactions are general to the meat from any species, but differences in the raw components, among them, the fatty acid profile, pro- and anti-oxidant content or in the meat structure, can affect the overall role of each reaction, and therefore, the final resultant aroma. In ruminants, precursors or aroma compounds can also arise from ruminal microorganisms or by a direct transfer from feeds [8].

The present review provides an updated overview of the chemical mechanisms and a discussion of the principal aroma compounds derived from those reaction or other sources in cooked meat, mainly focused in ruminant species.

## 2. Lipid Oxidation

During cooking, the oxidation of lipids, along with the Maillard reaction, contribute significantly to the formation in the meat of the typical, desirable aroma; however, lipid oxidation is also associated with the development of unpleasant rancid flavours in meat that has been stored, either raw or after it has been cooked [9]. Further than affecting the aroma characteristics, lipid oxidation can undermine greatly other aspects of quality, either through the changes generated in the lipid composition of cell membranes, the interactions between the products of oxidation and amino acids and various types of proteins, or the induction of other oxidative reactions (e.g., oxidation of cholesterol or fat-soluble vitamins). Thus, it can affect water-holding capacity, texture, and nutritional value, and promote the formation of toxic substances [10,11], but those aspects are not further discussed in the present review.

### 2.1. Mechanisms of Reaction

Within the rancidity mechanism, some of the products of the reactions cause the rate of rancidity to increase and, for that reason it is often referred to as auto-oxidation. However, in biological systems, an oxidative process requires a catalyst and the spontaneous formation of lipid radicals or the direct reaction of unsaturated fatty acids with molecular oxygen, is thermodynamically unfavourable [12]. While some aspects of the process are understood, many others remain unclear, particularly those related to the formation of some compounds in complex real models such as meat [13].

The rate of oxidation depends on the fatty acid composition, the concentrations, and the activities of pro- and anti-oxidants, the oxygen partial pressure, the structure and retained water in the meat, the method of processing (grinding, packaging), and the conditions in which the meat is stored (temperature, lighting) and cooked (method, temperature, and duration) [14,15]. Lipid oxidation has a primary and a secondary phase.

#### 2.1.1. Primary Phase

Initiation involves the removal of hydrogen from a methylene group, typically in a *cis* double-bond pair of an unsaturated fatty acid (bis-allylic hydrogen) [16] which forms a lipid radical (L•) that can be rearranged [9]. Polyunsaturated fatty acids (PUFA), that have two or more double bonds, are more prone to oxidation than are the fatty acids that have one or no double bonds because in the firsts the hydrogen can be more easily removed due to the formation of a stable allylic radical in which the electrons are delocalized over three carbon atoms [17]. When the degree of unsaturation of the fatty acid is higher, the induction period is reduced and the relative rate of oxidation increased [15]. The mechanism that initiates lipid oxidation is not well understood and remains the subject of some dispute. The decomposition of endogenous species (e.g., H_2_O_2_), radicals (O_2_^−^, LOO^•^, HO^−^, NO^−^), exogenous species (O_2_, O_3_), oxides (NO*_x_*^−^, SO_3_^−^), or agents (ultraviolet radiation, ionizing radiation, heat) might initiate the oxidative degradation of lipids [12]. Nevertheless, most biological and food studies of lipid peroxidation blame transition metal ions as rancidity initiators (Fe^n+^, Cu^n+^, *etc*.), and it is generally accepted that iron is pivotal in catalyzing oxidative processes that occur in tissues [12].

The lipid radical (L^•^) can react with molecular oxygen or oxygen derivatives substances such as O_2_^−^, HO^−^, H_2_O_2_^−^, to form a peroxyl radical (LOO^•^). This new radical reacts in turned with a triglyceride or a free fatty acid to produce hydroperoxide (LOOH) and a new free radical, which can reinitiate the process [16]. The propagation is relatively slow; consequently, hydrogen abstraction is selective for the most readily extractable hydrogen in an unsaturated fatty acid, and not from saturated fatty acids, especially when the reaction occurs at low temperatures [15]. The self-propagating reaction chain stops when an inactive substance is formed, e.g., when radicals are combined between them, or with a hydrogen or an electron donated from vitamin E, for example [16], or when the radical combines with other antioxidants or non-lipid molecules, e.g., proteins.

#### 2.1.2. Secondary Phase

**Scheme 1:**
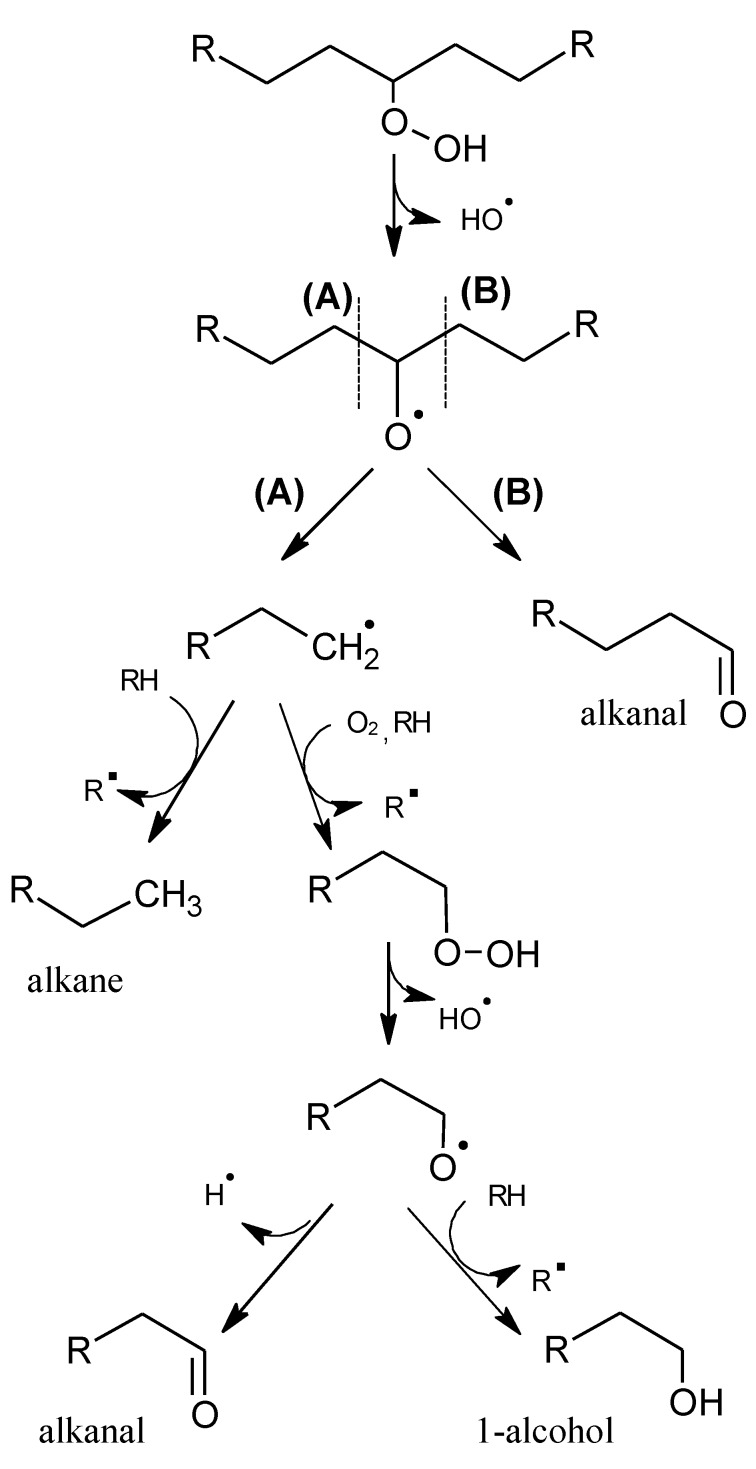
Oxidation of a saturated hydroperoxide. Adapted from [9], by permission of *Taylor & Francis*.

The secondary phase includes the decomposition of hydroperoxides and involves a complex series of reactions, e.g., the volatile aroma compounds. The processes are accelerated by heat, light, organic iron catalysts, and traces of metal ions, especially copper and iron. Initially, there is a homolysis of the hydroperoxides, which creates hydroxyl (^•^OH) and alkoxy (R-O^•^) radicals, continuing along the cleavage (β-scission) of the fatty-acid chain adjacent to the alkoxy radical (A or B, Scheme 1). The type of volatiles generated depends on the alkyl chain of the hydroperoxide and the position where cleavage occurs. If the alkyl group is saturated and the cleavage occurs in the alkoxy B radical, the result is a saturated aldehyde, but if the cleavage occurs in A (Scheme 1) the result is an alkyl radical that can produce an alkane or, alternatively, it can react with oxygen and produce other hydroperoxides. The latter can decompose in the same way (homolysis) and produce a stable, non-radical product such as an alcohol or aldehyde.

**Scheme 2:**
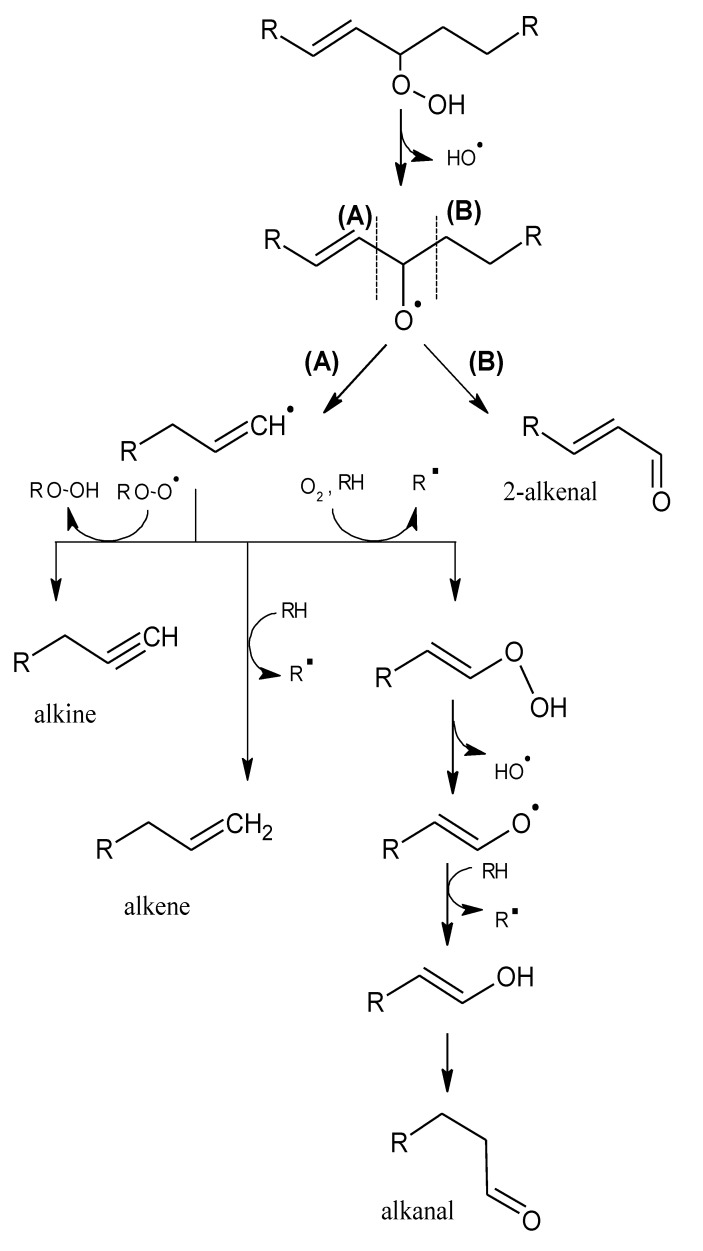
Oxidation of a monounsaturated hydroperoxide. Adapted from [9], by permission of *Taylor & Francis*.

**Scheme 3:**
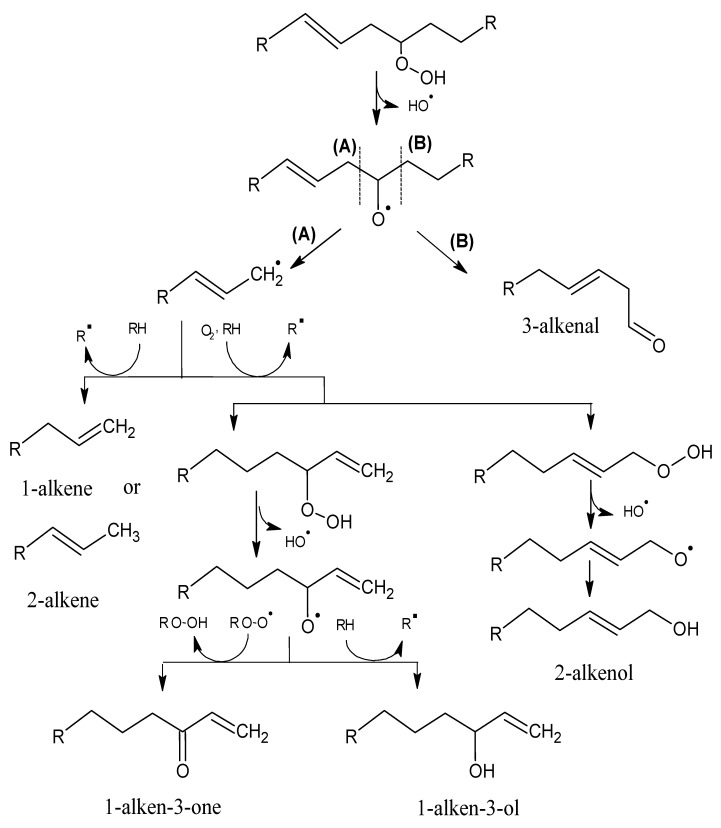
Oxidation of a monounsaturated hydroperoxide that has an allyl group. Adapted from [9], by permission of *Taylor & Francis*.

**Scheme 4:**
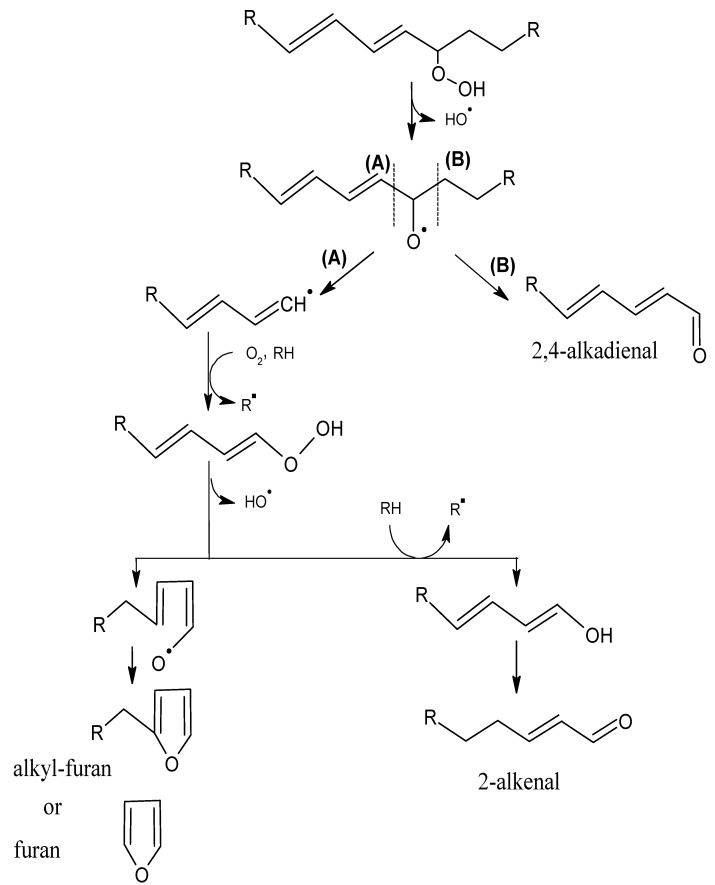
Oxidation of a polyunsaturated hydroperoxide. Adapted from [9], by permission of *Taylor & Francis*.

Alkyl chains that have one or more double bonds can produce analogous compounds that contain double bonds, although the ultimate number of products is greater because the unsaturated chains can be further oxidized [18]. A double bond in the alkyl chain adjacent to the alkoxy radical can generate alkenes and alkenals, with the double bond in position 2 (Scheme 2). If the double bond is separated from the alkoxy radical by a methylene group (allylic system), a greater variety of compounds can be formed (Scheme 3); e.g., vinyl alcohols and ketones, which have very low odour thresholds [15,19]. Hydroperoxides that contain a diene (two double bonds) can form alkadienals and alkylfurans (Scheme 4).

**Scheme 5:**
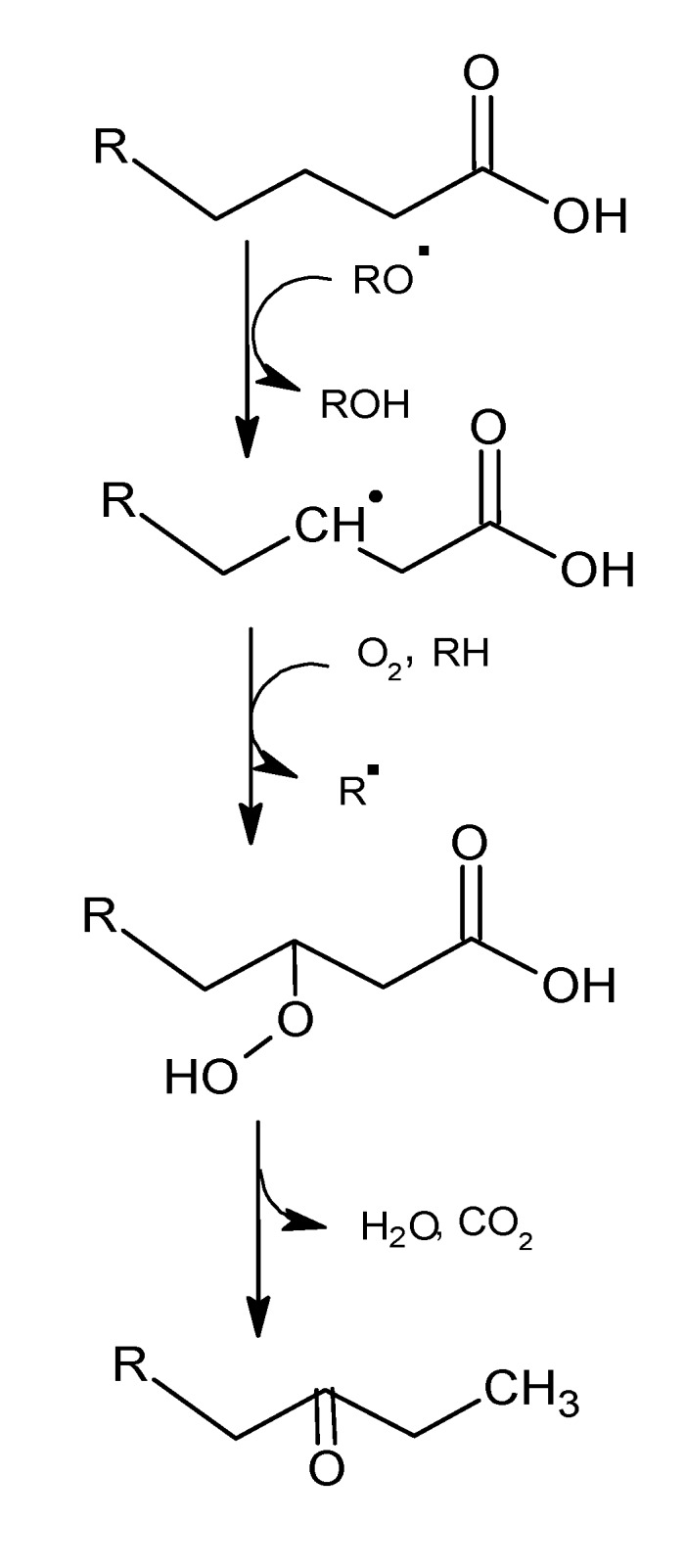
Hypothesis for the formation of methyl ketones. Adapted from [15], by permission of *Springer*.

High temperatures can facilitate the oxidation of saturated fatty acids. The volatile compounds generated by the thermal oxidation of tristearin, where aldehydes and methyl ketones account for 74.5% of the total profile by weight, are shown in Table 1. A hypothesis for the formation of methyl ketones, which involves the thermal β-oxidation of the saturated fatty acid and a decarboxylation, is shown in Scheme 5.

**Table 1:** Volatile compounds produced by heating tristearin to 192 °C. From [9], by permission of *Taylor & Francis*.

Homologous series	Number of carbons	Major compounds
*Aldehydes*	3–17	hexanal heptanal octanal
*Methyl ketones*	3–17	2-heptanone 2-nonanone 2-decanone
*Acids*	2–12	hexanoic acid pentanoic acid butanoic acid
*Hydrocarbons*	4–17	heptadecane nonane decane
*γ-Lactones*	4–14	γ-butyrolactone γ-pentalactone γ-heptalactone
*Alcohols*	4–14	octanol nonanol decanol

### 2.2. Aroma Compounds Derived from Lipid Oxidation

Although only a small proportion of the fatty acids in food are oxidized, it can be sufficient to alter significantly the flavour [15]. Aldehydes and ketones are the main aroma substances derived from lipid oxidation; however, hydrocarbons (alkanes, alkenes, and alkylfuranone) and alcohols, mainly vinyl alcohol, also play a role [15]. Other strong aromas derived from the thermal oxidation of lipids are lactones: γ-heptalactone [4,5-dihydro-5-propyl-2(3*H*)-furanone] and γ-octalactone [4,5-dihydro-5-butyl-2(3*H*)-furanone], which influence favorably the aroma of shallow-fried beef [20].

The degree of polyunsaturation in intramuscular fat is important because it can determine the overall concentrations of the volatiles derived from lipid oxidation [21]. As mentioned above, susceptibility to oxidation increases significantly with an increase in the number of double bonds in the fatty acids. The profile of the fatty acids can influence the types of aroma compounds derived, consequently, differences in fatty acid composition between species (Table 2), could explain in part the species-specific aroma. Even in ruminants, where a high percentage of lipids of the feedstuffs can be hydrolyzed and hydrogenated in the rumen, some can escape and be incorporated in animal tissues, reflecting in part what the animal had ingested, and, therefore, the aroma of the meat can be also affected by animal feeding [22].

**Table 2:** Fatty acids composition (%) triacylglycerol (neutral lipids) and phospholipids (polar lipids) of the *longissimus* muscle in pigs, sheep and cattle.

	Neutral lipids			Polar lipids		
Fatty acid	Pigs	Sheep	Cattle	Pigs	Sheep	Cattle
14:00	1.6	3.0	2.7	0.3	0.4	0.2
16:00	23.8	25.6	27.4	16.6	15.0	14.6
16:1 *n - 7*	2.6	2.2	3.5	0.8	1.5	0.8
18:00	15.6	13.6	15.5	12.1	10.4	11.0
18:1 *n - 9*	36.2	43.8	35.2	9.4	22.1	15.8
18:2 *n - 6*	12.0	1.5	2.3	31.4	12.4	22.0
18:3 *n - 3*	1.0	1.2	0.3	0.6	4.6	0.7
20:4 *n - 6*	0.2	nd	nd	10.5	5.9	10.0
20:5 *n - 3*	nd	nd	nd	1.0	4.1	0.8

nd: no detected. From [23], by permission of *Elsevier*.

### 2.3. Rancidity in Raw Meat

Lipid oxidation is one of the major causes of a reduction in the shelf life of meat products, mainly because of the formation of disagreeable aroma substances. Often, the negative aspects of lipid oxidation are referred to as *rancidity*. Subsequently to the slaughtering of the animal, all of the biochemical changes that occur in the muscles favor the pro-oxidative factors against the antioxidant protection [11]. The cessation of the circulation of blood and nutrients, a reduction in pH, the loss of the function of antioxidant enzymes, the degradation of muscle proteins by Ca-dependent proteinases, the destruction of the compartmentalization of cells, and the release of iron are examples of those changes [24].

Oxidation occurs even in frozen raw meat. Only an oxygen-free environment can prevent the reaction, but trace amounts of O_2_ are present even immediately after the product is vacuum-packed, and no commercial packaging is entirely impervious to oxygen [25]. However, vacuum packaging is used widely for the storage and distribution of cuts of meat because it limits lipid oxidation considerably [26]. In vacuum packed and frozen beef steaks from the *longissimus thoracis* muscle of the Morucha breed, significant levels of lipid oxidation do not occur until after 90 days in storage, and these levels do not have a negative effect on the assessment by a sensory panel [27].

Unfortunately, the purple appearance of vacuum-packed meat is not readily accepted by most consumers and, therefore, in retail markets, meat is displayed in oxygenated environments [26], despite the likelihood of oxidation. If rancidity is not controlled and unpleasant odours are formed in stored meat, they can persist after the meat is cooked [28]. Interesting finding were described by Zakrys and others [29], where beef steaks stored in packs with high oxygen content, even with high rancid notes, were preferred over less oxidized steaks, maybe due to the familiarity of panelists of oxidized flavors below a certain threshold level.

### 2.4. Lipid Oxidation during Cooking

Generally, it is assumed that the lipid oxidation that occurs when meat is cooked creates pleasant aromas, unlike what occurs in the raw meat. Although, if the fat is subjected to prolonged heating, disagreeable aroma compounds can be created [15].

The reaction mechanism of thermal lipid oxidation, which occurs during cooking, is similar to what occurs when meat is stored at low temperatures. Differences in the rate and magnitude of the processes, coupled with the interactions with other products of the reactions that occur during cooking might be the causes of the differences in the aroma profiles [30].

The cleavage of the fatty acids is less selective at higher temperatures. Thermal oxidation in the cooking process can promote the degradation of saturated fatty acids that does not occur at low temperatures, and the first products of oxidation (hydroperoxides) are degraded quickly [28]. Furthermore, high temperatures increase the concentrations of the hydroperoxides that have an (*E*),(*E*)-diene system, at the expense of those that have an (*E*),(*Z*)-diene system [31].

The volatile compounds of lipid origin dominate quantitatively the others in boiled, roasted or grilled pork, under mild temperature conditions [32], and that might be the case for ruminant meat. In addition, there are interactions among the various reactions; e.g., in roast beef, after an internal temperature of 77 °C is reached, the antioxidant effects of the products of the Maillard reaction can reduce lipid oxidation [19].

In a study performed with meat from 6-year old sheep, the amount of lipid oxidation derived aroma compounds present in raw were similar than in pressure-cooked meat [6], and therefore, differences in the release of compounds from the meat matrix might explain the sensorial distinction between raw and cooked meat aroma. In cooked meat, aldehydes can interact with proteins and, thus, are not released into the headspace [33]. Furthermore, the relatively high viscosity of meat cooked at high temperatures might block the movement of molecules into the headspace [33]. Finally, other not lipid-derived volatile compounds, can hinder the detection of those compounds derived from lipid oxidation [33].

### 2.5. Unpleasant Aromas in Meat after Reheating

Once meat is cooked, if stored in the refrigerator at about 4 °C, and especially after reheating, lipid oxidation can cause rancid flavours in just two days, usually named warmed over flavours [34]. The aforementioned authors, using different methods of extraction and analysis to identify potentially important odorant compounds and after performing a sensorial evaluation of the aromas in meat models, concluded that the increase of hexanal and *trans*-4,5-epoxy-(*E*)-2-decenal and the reduction of two furanones (sotolon and furaneol) were the causes of the changes in the aroma quality of reheated meat. The last two compounds are responsible for desirable flavours in the meat, which might interact with lipid radicals.

In ground beef cooked and stored for 4 days at 4 °C, rancidity products, primarily saturated aldehydes and, especially, hexanal, were significantly increased [19]. High concentrations of antioxidants, such as vitamin E in the diet of the animals, whether consumed naturally with pasture or added to concentrates, controls the oxidation and the development of unpleasant flavours in meat that is cooked, stored and reheated [35].

The addition of aldehydes to a mixture of ground beef and water reduced the typical agreeable aromas of the meat, while rancid notes (paint and herbal) can be detected [33]. However, the aldehydes and other compounds derived from lipid oxidation form part of the typical aroma profile of the meat without deterioration of the flavour. There is a threshold above which the aroma becomes disagreeable but, below this threshold, the products of oxidation can contribute positively to the aroma of meat and other foods [15]. The detection thresholds that probably indicate deterioration in the aroma quality of beef, measured in a meat-water model [33], are the following: 2.67 ppm pentanal, 5.87 ppm hexanal, 0.23 ppm heptanal, 7.87 ppm (*E*)-2-hexenal, 4.20 ppm (*E*)-2-octenal, and 0.47 ppm (*E,E*)-2,4-decadienal.

### 2.6. Phospholipids and Triacylglycerols in the Flavour of Meat

Meat contains fat that cannot be removed by the consumer before or after cooking. The intramuscular fat is composed of polar lipids or phospholipids, components of cell membranes; and other apolar lipids, triacylglycerols, sometimes visible inside the meat (marbling). The extraction using petroleum spirits of non-polar or neutral lipids in beef do not markedly affect the aroma of meat of the cooked residual material [36]. However, the extraction of neutral and polar lipids (using cloroform/methanol), significantly reduces the aroma of the meat. 

One of the reasons why phospholipids have a greater influence on the aroma of meat than triacylglycerols is that the former are composed of a greater proportion of polyunsaturated long-chain fatty acids, and, therefore, are more susceptible to lipid oxidation (Table 2). That is especially true for sheep meat and beef; in pork, triacylglycerol oxidation can also be important, because of its higher proportion of polyunsaturated fatty acids compared to that in the meat of ruminants (linoleic acid, C18:2 ***n-6*** and arachidonic acid, C20:4 ***n-6***) [23].

Another reason for the greater influence of membrane phospholipids on the oxidation of lipids is that the polar phase is in contact with the main catalysts in the reaction (oxygen, metals, and peroxidases). In addition, usually, phospholipids are the main substrates of lipolysis, and free fatty acids are more easily oxidized. On the other hand, a type of phospholipid that is part of cell membranes, plasmalogens, can contain an aldehyde (12-methyltridecanal) formed by rumen microorganisms [37,38], which might be one of the main compounds that contribute to the aroma of boiled or stewed beef (Table 3).

The apparent lesser contribution of triglycerides in lipid oxidation does not mean that they do not participate actively in the flavour of cooked meat. Adipose tissue can be a reservoir of aroma compounds (e.g., terpenes, indoles, phenols, lactones) or their precursors (e.g., β-carotenes), soluble in lipids, which can come directly from the animal’s diet or from the rumen [8,39,40]. In addition, when meat is consumed, fat can affect the release or retention of aroma compounds, which can alter the sensory evaluation of the product [41]. In sheep meat, triglycerides appear to be particularly important in the aroma, where even in the uncooked fat the odour of “sheep” is evident [42]. Branched-chain fatty acids (BCFAs) of medium length, especially 4-methyl/ethyl-octanoic/nonanoic acids that are deposited in the triacylglycerols, are involved in the flavour of sheep and goat meat [43,44,45]. More details of those compounds are exposed afterwards.

## 3. Thiamine Degradation

Model systems have shown that thermally degraded thiamine (vitamin B_1_) produces a multitude of sulphur compounds, e.g., thiols, sulphides and disulphides [46,47]. Some of those compounds at low concentrations in themselves smell like cooked meat and some of them contribute significantly to the aroma of cooked meat [48]. 2-Methyl-3-furanthiol and 2/3-mercapto-3/2-pentanone are examples of important volatiles that can arise from the degradation of thiamine [49]; however, these sulphur compounds can be derived from other pathways, e.g., the Maillard reaction between cysteine and ribose, the Strecker reactions of sulphur amino acids and the interactions among them [49].

Thiamine is a hydrosoluble vitamin that is very susceptible to thermal degradation, where almost 100% can be lost in some processing methods [50]. The concentrations of thiamine (per 100 g) in raw beef, lamb, chicken, and pork are approximately 0.08–0.11 mg, 0.17–0.18 mg, 0.23 mg, and 0.81–0.88 mg, respectively [50,51,52,53]. Among other factors, the duration, temperature, type of cooking, the presence of other ingredients and pH influence the losses of thiamine. As an example, in pork roasted to an internal temperature of 72 °C, the vitamin is retained in the meat juice expelled but not thermally degraded [54].

**Scheme 6:**
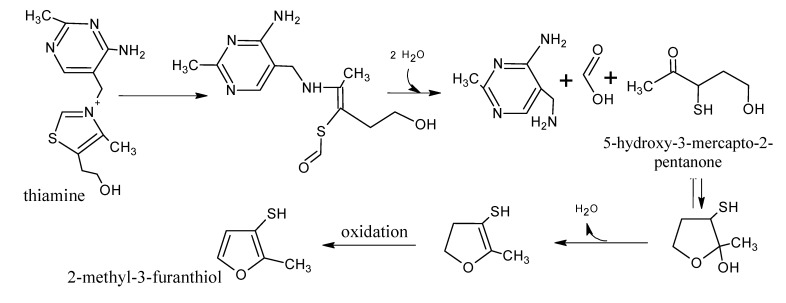
Formation of 2-methyl-3-furanthiol from thiamine via 5-hydroxy-3-mercapto-2-pentanone. Adapted from [57], by permission of *American Chemical Society*.

Using model systems, Grosch, Zeiler-Hilgart, Cerny, and Guth demonstrated that, even in concentrations as low as those found in real beef, thiamine was more efficient in producing 2-methyl-3-furanthiol than cysteine/ribose [55]. The compound 2-methyl-3-furanthiol has a very low olfaction threshold (0.007 µg/kg in water) and is present in lamb, pork, chicken, but especially, in cooked beef [48]. In addition, it is a key component in simulated beef flavourings [56]. In Scheme 6, an outline of the formation of 2-methyl-3-furanthiol from the degradation of thiamine and the intermediary 5-hydroxy-3-mercapto-2-pentanone is shown [57]. Similar pathways have been proposed for other thiols and mercaptoketones that contribute to the aroma of meat [58].

**Table 3:** The most important carbonyl compounds * in beef and sheep/lamb meat identified using GC-O.

Reference	[31]	[37]	[20]	[59,60]	[61]	[62]		[34] ^a^	[34] ^b^	[6]		[63]	[64]		Origin probable ^e^
Type of meat	Beef	Beef	Beef	Beef	Beef	Beef		Beef		Sheep		Lamb	Lamb		
Cooking method	Boiled	Boiled	Fried	Roasted	Fried	Stewed (juice)		Fried patties		Raw	Boiled	Grilled	Grilled		
Extraction method ^c^	SD ^dc^	SD ^ac^	DHS ^dc^	DHS ^dc^	DHS ^ac^	SHS ^ac^	SD ^ac^	SD ^ac^		SD	SD ^ac^	DHS ^ac^	Mouthspace	DHS ^dc^	
GC-O analysis ^d^	DA	DA, CA	DA	DF	DF	DA		DA		DA		MF	MF	MF	
hexanal					2	2			3						LO
heptanal			3												LO
octanal							3							3	LO
nonanal			2	4											LO
decanal												1			LO
*(E*)-2-heptenal												2			LO
*(E*)-2-nonenal	1	1	1	3	4			2	2	3	2		2		LO
*(Z*)-2-nonenal							4					3			LO
*(Z*)-3-nonenal								3	4						LO
*(E*)-2-decenal			4												LO
*(Z*)-2-decenal													3	1	LO
*(E,E*)-2,4-heptadienal												1			LO
*(E,E*)-2,4-nonadienal					4			4		3					LO
*(E,E*)-2,4-decadienal	1					2	2	2		2	2	4	1	2	LO
4,5-epoxy-(*E*)-2-decenal							4			1	1				LO
ethanal acetaldehyde						1									SR
methional	1	3	1	1		2	1	1	1						SR
3-methylbutanal					1	2									SR
phenylacetaldehyde	2														SR
12-methyltridecanal		3					1								B
1-octen-3-one	2	1					4		4	3	2				LO
1-nonen-3-one				4											LO
3-mercapto-2-pentanone		2													TD
2,3-butanodione				2	1	2									B, MR
2,3-pentanodione					3										MR
(*Z*)-1,5-octadien-3-one										2	2				LO
β-ionone	1														CD

* 1,2,3, and 4 represents the hierarchy of the carbonyl compounds within each reference; ^a^ after cooking; ^b^ cooked and stored 48 h at 4 °C; ^c^ SD: solvent distillation, DHS: dynamic headspace; SHS static headspace, ^dc^: during cooking, ^ac^: after cooking; ^d^ DA: aroma extract dilution analysis, CA: aroma extract concentration analysis, DF: detection frequency; MF: modified frequency; ^e^ LO: Lipid Oxidation, SR: Strecker Reaction, MR: Maillard Reaction, TD: Thiamine Degradation, B: Bacterial Action, CD: β-carotene Degradation.

However, adding 4-fold the natural concentration of thiamine to raw beef, no differences in the flavour of the cooked meat were found [65]. It seems that for producing furanthiols and other impact aroma compounds, a higher concentration of available phosphates are needed [65]. Similar results were found in chicken, and only when the concentration exceed by far the actual values (450-fold), was possible to see an effect in the flavour [66]. In pork, since it contains more thiamine and seems to be more stable [67], this precursor might be more important [68] than in meats from other species, such as ruminants.

## 4. Strecker Reaction

**Scheme 7:**
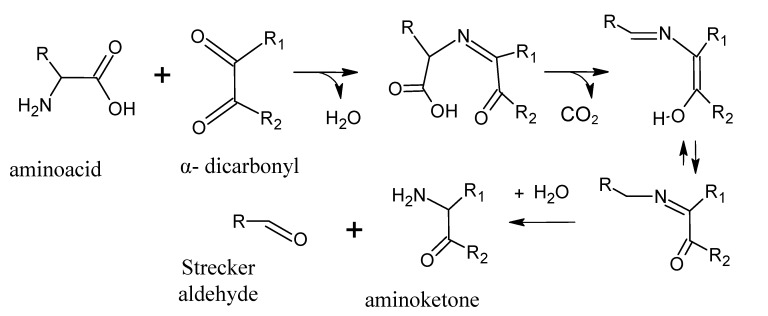
Strecker reaction. Adapted from [9], by permission of *Taylor & Francis*.

The Strecker reaction is often perceived as a reaction within the Maillard reaction [69] and, typically, the Strecker reaction involves the oxidative deamination and decarboxylation of α-amino acid in the presence of an α-dicarbonyl compound (Scheme 7). The products are an α-aminoketone and a Strecker aldehyde, which contain one carbon less than do the corresponding amino acid (Table 4).

**Scheme 8:**
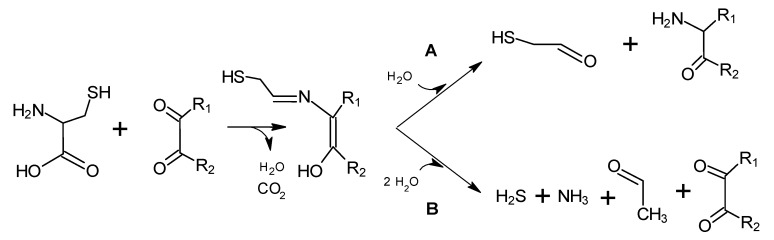
Degradation of cysteine. Adapted from [18], by permission of *Springer*.

**Scheme 9:**
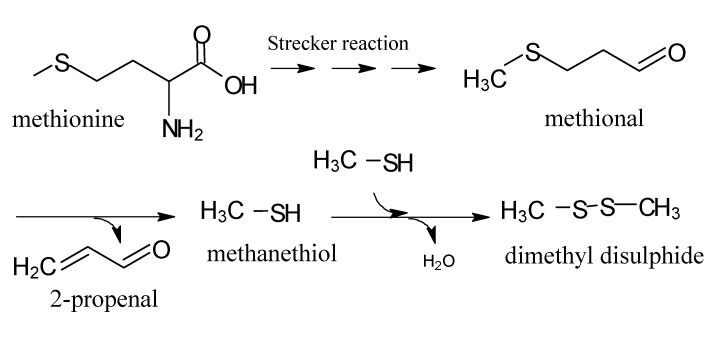
Degradation of methionine to methional, methyl mercaptan (or methanethiol) and dimethyl disulphide. Adapted from [15], by permission of *Springer*.

Cystein degradation is particularly important since produces intermediates implicated in the formation of very active compounds in meat flavour [18]. The process is outlined in Scheme 8, where from mercaptoiminoenol, mercaptoacetaldehyde and an aminoketone can be produced (Scheme 8A), or also hydrogen sulphide, ammonia, acetaldehyde, and a regenerated dicarbonyl (Scheme 8B). Another sulphur-containing amino acid, methionine, through the Strecker reaction produces methional, which has a low odour threshold and is important in the aroma of cooked meat. Methional breaks down rapidly [70] to 2-propenal and methanethiol, and the latter can form dimethyl disulphide (Scheme 9) and other sulphides [46]. Hydrogen sulphide, carbon disulphide, methanethiol, dimethyl sulphide, dimethyl disulphide and dimethyl trisulphide have been detected by gas chromatography-olfactometry (GC-O) in cooked beef [47,61,71].

**Table 4:** Strecker aldehydes detected by GC-O in cooked beef extractions ^a^, amino acid precursor, and odour threshold ^b^.

Strecker aldehyde	Structure	Amino acid precursor	Odour threshold
ethanal = acetaldehyde	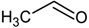	α-alanine/cysteine	25
propanal	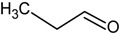	α-aminobutyric	-
2-methylpropanal	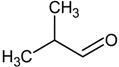	valine	2
3-methylbutanal	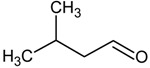	leucine	3
2-methylbutanal	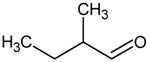	isoleucine	4
methional	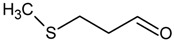	methionine	0.2
2-phenyletanal = phenylacetaldehyde	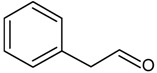	phenylalanine	4

^a^ [31,59,61,62]; ^b^ In µg/L, determined in water [15].

Strecker aldehydes also can be formed independently of the pathways established by the Strecker reaction, either directly from free amino acids or from Amadori products [69,72], or by microbial action [73]. On the other hand, Amadori or Heyns rearrangements of ammonia with reducing sugars can generate 2-aminocarbonyl compounds without the formation of Strecker aldehydes [69].

From the Strecker reaction, in the presence of glucose or generated α-dicarbonyls, the respective acids can also be formed; for example, acetic acid from alanine, 3-methylbutanoic acid from leucine or phenylacetic acid from phenylalanine [74]. Some acids, perhaps arising from the Strecker reaction, have been identified as important aromas in cooked beef [37] and chicken [75]. Using GC-O the acids 2- and 3-methylbutanoic have been detected in lamb fat [76], which might have been formed by microbial action in the rumen [77]. On the other hand, acids, aldehydes and esters derived from the degradation of amino acids, might be indicative of meat contaminated by microbial action during the storage and distribution of meat products [73,78].

## 5. Maillard Reaction

The Maillard reaction is key in the formation of most of the aroma compounds recognized in cooked meat [79]. It includes a very complex series of reactions that begin with the condensation between the carbonyl group of a reducing sugar and a free amino group [80]. Although the Maillard reaction can occur at refrigeration temperatures, the reaction rate increases significantly when the cooking temperature is increased [9]. By contrast, caramelization or the pyrolysis of proteins, needs high temperatures (100–200 °C) to develop [18]. It has also been observed that Maillard reaction is affected by water activity and that any subsequent reaction pathways depend heavily on pH. The nature of the reacting compounds, e.g., type of sugar, amino acid, or protein, the presence of substances that can interfere with the reaction (e.g., carbonyl compounds derived from lipid oxidation), and even the matrix can influence the profile of the resulting aroma compounds.

### 5.1. Mechanisms of Reaction

**Scheme 10:**
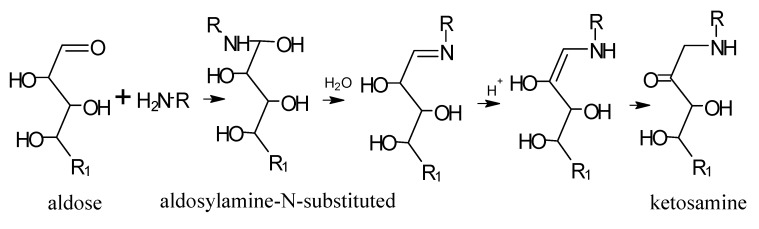
Amadori rearrangement. Adapted from [81], by permission of *The Royal Society of Chemistry* (http://dx.doi.org/10.1039/9781847550866).

**Scheme 11:**
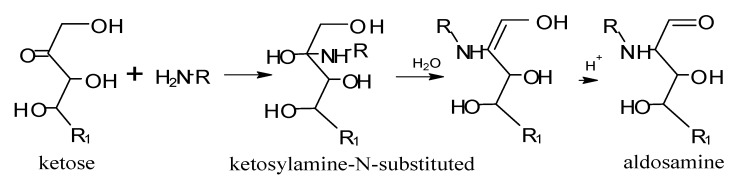
Heyns rearrangement. Adapted from [81], by permission of *The Royal Society of Chemistry* (http://dx.doi.org/10.1039/9781847550866).

If the initial reducing sugar is an aldose monosaccharide that has an aldehyde group, e.g., ribose or glucose, it forms an aldosylamine-N-substituted, which is rearranged to form an intermediary amadori (Scheme 10). If the reducing sugar is a ketose monosaccharide that has a ketone group, e.g., ribulose or fructose, Heyns rearrangement of ketosylamine-N-substituted and the corresponding Heyns intermediary are produced (Scheme 11).

**Scheme 12:**
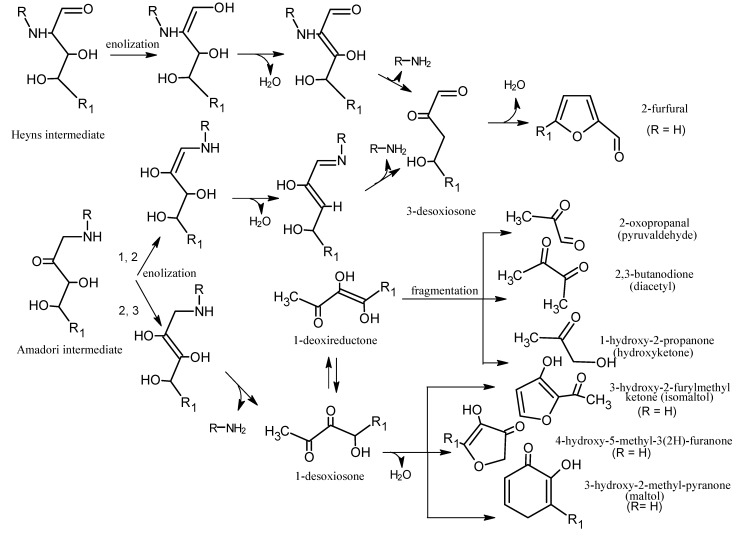
Decomposition of Heyns and Amadori intermediates. Adapted from [79], by permission of *Springer*.

Next, the Amadori/Heyns products are degraded, which leads to dehydrations and deaminations that produce carbonyl compounds such as deoxysones and deoxyreductones. Then, many types of reactions are produced (e.g., dehydrations, fragmentations, cyclizations, and polymerizations) in which the amino groups can participate again, for example, through the Strecker reaction [82]. In Scheme 12 a general path of some of those reactions is outlined.

The dicarbonyl compounds, furfurals and furanones, in addition to the Strecker aldehydes, ammonia, and hydrogen sulphide, can influence the aroma of cooked meat by themselves, but also are key intermediaries to other important components in the flavour of meat [18]. Among them, H_2_S and NH_3_ are the most reactive, and they can interact with the products of lipid degradation and, thus, affect the relative production of heterocyclic compounds that are derived from the Maillard reaction [18].

**Scheme 13:**
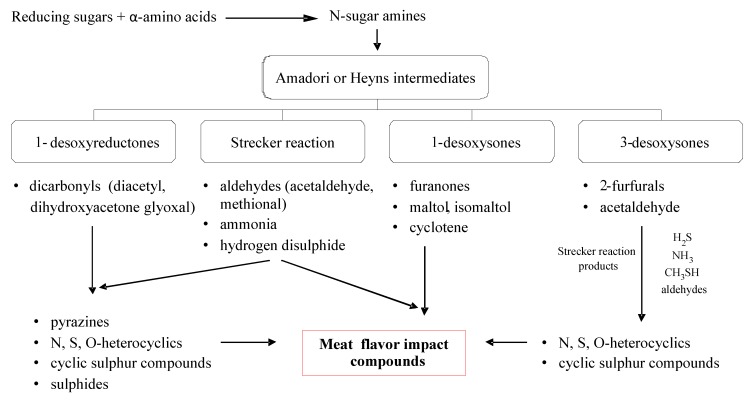
Formation of impact aromatic compounds in meat from the Maillard reaction. Adapted from [79], by permission of *Springer.*

Scheme 13 outlines the formation of important aroma compounds in meat that are derived from the Maillard reaction. Compounds formed from deoxyreductones can react with the degradation products from the Strecker reaction to form pyrazines, heterocyclic compounds with nitrogen and sulphur (e.g., thiazoles and thiazolines), cyclic sulphur compounds and mono-sulphides. The precursors formed from 1-deoxysones can interact with the products of the Strecker reaction to produce numerous aromatic compounds (e.g., furanones, thiophenones, furanthiols).

## 6. Aroma Compounds of Cooked Meat

Through the chemical reactions mentioned and their interactions, many types of aromatic compounds (e.g., carbonyl, sulphur, and pyrazines) are formed. The derivatives of α-linolenic acid can reduce the concentrations of the compounds that give meat its typical aroma (thiophenes, furans) through their interactions with the products of the Maillard reaction, and they are more reactive than the products of the degradation of *n*-6 and *n*-9 fatty acids [83]. Other aromatic compounds can come from the metabolism of the rumen, the animal itself, or directly from the food consumed by the animal, all of which accumulate in the fatty tissue in meat.

An aromagram based on a meta-analysis of recent literature on GC-O data revealed that furaneol and methional were of greater relative importance in beef aroma compared to lamb/sheep aroma, while sheep (but not lamb) meat aroma might be mostly defined by 4-ethyloctanoic acid [5]. Other study, with beef/vegetable based gravy versus pork/vegetable based gravy indicated that most of the aroma compounds were identical, although different in aroma importance. The key odorant 12-methyl-tridecanal was only detected in GC-O of the beef/vegetable gravy [84]. Those and other studies show that the distinction in the meat aroma from different animal species is not necessarily due to an aroma compound profile qualitatively different (few compounds are present only in one species), and therefore, the sensory discrimination could be rather be due to differences in the quantity of a particular compound with implication in the perceived aroma. 

It has to be taken into account that subtle quantitative differences might greatly affect the perceived aroma and that sometimes a given compound can smell different according to their concentration, since higher concentrations of a scent can stimulate the use of more types of receptors [85]. Furthermore, the combination of two odorants can activate neurons in the cerebral cortex that are not activated by the individual components, which might explain why, for example, ethyl butyrate smells fruity and diacetyl smells buttery, but a mixture of the two smells like caramel [86]. Inhibition, addition or synergism might occur between the odorants that have been capable to release from the matrix; hence, more reconstitution studies are necessary to conduct to fully understand the role of the individual compounds in the overall aroma perception and acceptability.

Following the outstanding aroma compounds detected by GC-O studies in meat from beef and lamb/sheep meat and their probable origin and overall role in the cooked meat are presented.

### 6.1. Branched-Chain Fatty Acids

Some medium-length, BCFAs were considered key in the aroma of sheep and goat meat. In a sensory study performed with trained panelists and consumers from New Zealand (familiar with sheep meat) and Japan (not habituated to sheep meat), cooked lean beef samples was spiked with BCFAs (and skatole) at three levels (none, low, high) [87]. New Zealanders liked equally meat spiked with none or low BCFAs, whereas Japanese disliked any BCFA presence. High levels of BCFAs were associated with barnyard/milky/sour/sheepmeat flavours and it was suggested that reductions in BCFAs (and also skatole) may improve the acceptability of sheep meat in Japan, and perhaps other North Asian markets.

In lambs, branched-chain fatty acids accumulate in subcutaneous fat, primarily; and its importance in lean lamb is not so clear because concentrations are below the detection threshold [43], whereas intramuscular fat contains higher concentration of 4-methyl-/ethyloctanoic acids than other adipose tissue in 6 year old sheep [6]. In addition, the concentrations of these fatty acids vary depending on the diet, sex, and age of the animal [44,88]. The concentrations of those fatty acids in fat are known to be higher in grain-fed than in pasture-fed lambs because these fatty acids can be synthesized from propionate, which is formed in higher quantities in the rumen of lambs that received a concentrate-based diet [44,89].

An Aroma Extraction Dilution Analysis (AEDA) had shown that 4-ethyloctanoic acid play an important role as odorant in raw and pressured cooked meat from 6 year old sheep [6], even though in other GC-O studies involving headspace extractions, none of the medium length branched, 4-methyl-/ethyloctanoic/nonanoic, fatty acids were detected, neither in meat nor in fat of lambs [63,64,76,90]. Those compounds were not detected neither in the volatile analysis performed with goat meat [91,92]. Its low concentration in lean meat from young animals [43], as oppose as the case of older animals, might explain the GC-O results found. Other aspects should be taken into account, as the low volatility of these acids or the fact that under mild cooking/extraction conditions, hydrolysis might not be considerable and so these fatty acids, not completely released from triacylglycerols [44,76,90].

Branched fatty acids with lower molecular weights than those noted above can be formed by the deamination and metabolism of valine, leucine and isoleucine in the rumen. The formation of those compounds is favored by some pasture diets and might be involved as well in the aroma of the ruminant meat [39].

### 6.2. Carbonyls

Table 3 shows hierarchically the most important carbonyl compounds in bovine and ovine meat identified using GC-O. On the other hand, the table also allows analyzing that cooking method, the way in which the volatile compounds are extracted, and the type of GC-O analysis performed can affect which of the aromas are detected as important, and should be taken into account when comparing data from different studies. In the extraction of the static headspace, for example, ethanal is one of the main aromas, but this is not the case when the extraction involves a solvent distillation method [62]. 

Among the carbonyl compounds derived from lipid oxidation, (*E*)-nonenal and (*E,E*)-2,4-decadienal stand out in both species. Those compounds and (*Z*)-2-decenal are among the most important compounds in the mouth-headspace of consumers of grilled lamb [64]. It is noteworthy that those compounds not only evoke rancid odours, they also evoke meaty odours [59,63,64].

In addition, hexanal and 1-octen-3-one become more important, aromatically, in cooked meat patties that is refrigerated for 2 days, while alkadienals are reduced ([34], Table 3), perhaps, because of successive oxidation reactions. However, comparing raw and cooked from sheep meat, no differences in the hierarchy of the carbonyl compounds is observed ([6], Table 3), so it seems that lipid oxidation take place before cooking and thermal lipid oxidation is of minor importance, or that interactions between reactions took place while cooking. In the mentioned study, total compounds present were analysed and not only the ones released from the matrix, and therefore, the actual aroma hierarchy found can change (more discussion is presented in Section 2.4).

The Strecker reaction, Maillard reaction, the degradation of thiamine, bacterial action, and the degradation of β-carotene are other mechanisms through which important aromatic aldehydes and ketones are formed in meat. Methional, a carbonyl but at the same time, a sulphur compound, is more a beefy than a lamb/sheep aroma. The aldehyde, 12-methyltridecanal, arising from the phospholipids (see Section 6.2) is important according GC-O studies in boiled and stewed beef, but not through other methods of cooking, e.g., frying or grilling (Table 3), in which the meat is subjected to high temperatures for a relatively short time can reduce the release of 12-methyltridecanal from phospholipids [38]. However, in a meat juice model system, this compound did not significantly contribute to the overall aroma [47]. 

### 6.3. Indoles, Phenols, Terpenes and Lactones

The concentrations of indole and 3-methylindole (skatole) in sheep fat can be associated with unpleasant aromas [44,76], although these compounds do not appear to be important in beef [93]. Microbial activity in the rumen metabolizing the amino acid tryptophan is largely responsible for the formation of indole and skatole in ruminants. Species differ in the importance of skatole, perhaps because of differences in concentrations [76] or because skatole acts synergistically with other aromatic compounds in sheep meat [93].

The aromatic phenolic compounds in the meat of ruminants can come directly from those present in plants or they are a product of the microbial fermentation of lignin or diterpenes [94]. In addition, they can be formed from the microbial metabolism of the amino acid tyrosine [39]. With cooking, hydrogen sulphide has the potential to act with phenols to form thiophenol, which might contribute to the aroma of sheep meat [94]. Recently, 2,6-dichlorophenol was identified by GC-O in lamb [64].

Terpenes are synthesized almost exclusively in the plant kingdom, thus, their presence in the meat and dairy products of ruminants might be an indicator of diets based on green forage [8]. In some studies, the concentration of 1-phythene, 2-phythene, neophytadiene, and β-caryophyllene in the adipose tissue of ruminants was more than ten times higher in the animals that were fed green forages than it was in the animals fed concentrates [8], although, the levels of β-gurjunene were higher in the adipose tissue of animals that were fed a concentrate diet [95,96]. Beyond that, although terpenes allow the discrimination of meat produced in the two types of feed systems, the impact that they have at the sensory level is not entirely clear. The intensity of the grass flavour detected sensorially in beef has been correlated, statistically, with the concentrations of several terpenes [97]. However, despite the significant differences found in the concentrations of 1-phythene (along with other volatiles non-terpenes) in the beef from animals fed grass silage and that from animals fed concentrates, differences in the sensory evaluations performed by a trained panel have not been observed [98,99]. Although, those compounds are individually present in low concentrations (relative to their detection thresholds), collectively, they might contribute with some aromatic notes to the meat.

Lambs finished on *Angelica archangelica*, rather than traditional pasture produced meat that had a different aroma and flavour (‘spicy’), mainly, due to a higher concentrations of terpenoid compounds such as β-phellandrene and α-pinene [100]. 

Lactones can be formed from hydroxy acids, which come from the oxidation of fatty acids that can occur in the rumen [101]. It has been suggested that the low pH in the rumen of cows fed concentrate facilitates the concentration of lactones in milk, although they come from α-linolenic acid [102], which might apply to meat. Lactones appear to be more concentrated in the fat of animals finished on concentrate than in those finished with on pasture or grass. That has been the case with γ-pentalactone, γ-butyrolactone, δ-tetradecalactone, and δ-hexadecalactone [96,97,103]; however, γ-butyrolactone, δ-decalactone, δ-dodecalactone are predominant when the animals are finished on pasture [97,104].

### 6.4. Pyrazines

The pyrazines 2,3-dimethyl-5-dimethylpyrazine and 2-ethyl-3,5-dimethylpyrazine are high impact aromas in roasted and fried meats [20,105], but they have not been detected by GC-O in boiled meat [37]. Aromatically important pyrazines are still being discovered, e.g., 2-isopropyl-3-methoxypyrazine in grilled lamb [64]. Reaction conditions such as moisture content, temperature, pH, and cooking time are important for the formation of pyrazines and, when those conditions are favorable, it is possible to find them in all types of meat [79]. In pork, the proportion of pyrazines among all of the volatiles detected in the headspace varies depending on the cooking method and the temperature at which it is cooked [32]. In grilled meat that is ‘well done’ almost 80 % of the volatile compounds are pyrazines.

In beef models, an increase in pH promoted the formation of heterocyclic compounds, e.g., thiazoles and pyrazines, whereas lower pH favored the formation of furanthiols and derived compounds [106]. The large quantity of non-protonated amino groups at high pH values leads to an increase in the availability of nitrogenous groups, which leads to an increase in the production of pyrazines and thiazoles [106].

Several mechanisms can be involved in the formation of pyrazines but, apparently, the Maillard reaction is the most important, particularly, if the Strecker reaction is included [79]. Most pyrazines are formed by the condensation of two α-aminocarbonyls, which forms dihydropyrazine, which oxidizes spontaneously to produce the corresponding pyrazine (Scheme 14A). α-Aminocarbonyl compounds can be derived from the Strecker reaction between an amino acid and α-carbonyl compounds, where the latter can be a product of the Maillard reaction or the caramelization of carbohydrates. Another pathway (Scheme 14B) involves the reaction between dihydropyrazine and a carbonyl compound, without the need for oxidation [107,108]. The nitrogen source does not necessarily involve an amino acid; for example, the source can be ammonia or urea [109,110]. In beef, chicken, and pork, urea concentrations of 41.4 mg, 7.2 mg, and 25.6 mg (per 100 g) have been respectively documented [110].

**Figure 14:**
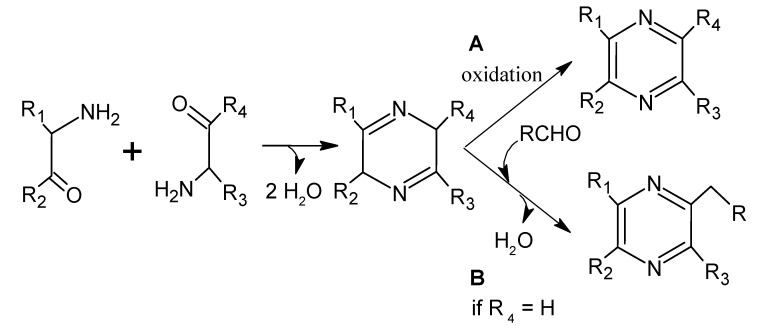
Schema of the formation of alkylpyrazines. Adapted from [108], by permission of *Elsevier.*

In one study, the addition of 0.5 % glucose compared to that present in beef muscle increased the concentration of pyrazines in cooked meat, but did not have an effect on the concentrations in the liver, where glucose is not a limiting factor in the formation of pyrazines [111]. Ovine meat has a reducing-sugar content that is lower than the concentrations in meat from other species [112], so that the addition of a minimal amount of sugar would probably increase the concentrations of pyrazines and other compounds derived from the Maillard reaction. The addition of xylose improves the aroma of sheep meat [113,114].

The maturation time of the meat and the animal’s diet can affect the natural concentrations of reducing sugars and free amino acids in muscle [115,116]; however, the differences in the volatile compounds in grilled meat are more closely associated with the effect of diet on the fatty acid composition of meat than with the effect on soluble aromatic precursors [117]. The natural concentration of sugars (primarily glucose and glucose-6-phosphate) should be at least double or threefold to be able to detect a change in the aroma of cooked beef [118].

The reactivity and type of amino acid present also influence the variety and amount of pyrazines produced [119]. The high reactivity of the presence of nucleophilic amino functions in the case of lysine and arginine, and the flexibility of glycine due to the absence of a side chain, might explain the higher quantity of pyrazines formed by the aforementioned amino acids in model systems [107]. On the other hand, the addition of the amino acid cysteine to liver, reduces the concentrations of pyrazines [111]. The actual matrix can influence the way in which the reactions occur [107].

Alternatively, the decarbonylation and dehydration of hyroxyaminoacids such as serine and threonine can form pyrazines without the use of a carbohydrate source precursor [120]. When meat is cooked at high temperatures, the pyrolysis of amino acids can be more important than the Strecker reaction [46]. On the other hand, fungi and microorganisms can produce pyrazines that have an unpleasant odour [15], e.g., the alkylmethoxypyrazines in ham produced by *Pseudomonas* [121].

Lipid oxidation can influence the formation of heterocyclic compounds, including the pyrazines. The aldehydes of lipid oxidation can react with ammonia to form non-volatile Schiff bases, which reduces the availability of NH_3_ for the synthesis of pyrazines [13]. Similarly, the reaction between H_2_S and the derivatives of lipid oxidation might explain the reduced availability of this key compound in the formation of heterocyclic sulphur compounds [13].

### 6.5. Sulphur Compounds

Undoubtedly, sulphur compounds play a fundamental role in the aroma of meat because they have very low detection thresholds [49]. Nevertheless, detection is difficult because they occur in very low concentrations, are very reactive, degrade easily, and many are still unknown, which makes it harder to control [49,60].

**Figure 1:**
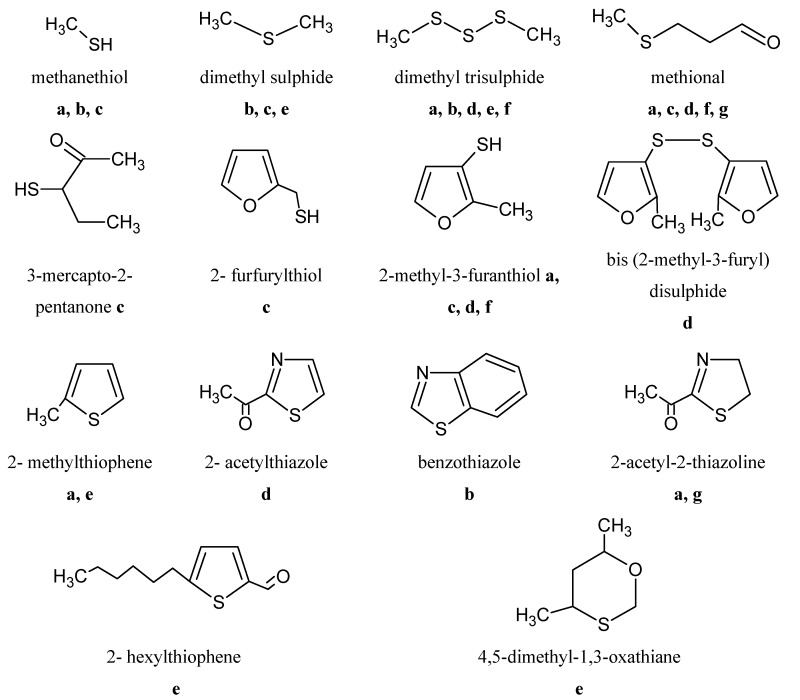
Sulphur compounds detected by GC-O in the aroma of beef and lamb.

Figure 1 outlines 14 aromatic sulphur compounds in beef and lamb. In one of those studies, using two-dimensional gas chromatography coupled with time of flight mass spectrometry (TOF-MS), 70 sulphur products have been found in the headspace of meat during cooking in the oven, of which 50 could be identified [60]. In the same study, using GC-olfactometry, six of those compounds were considered to have aromatic impact.

In beef: a [60], b [71], c [47], d [31]; In lamb: e [88], f [63], g [64].

Sensory omission experiments have demonstrated the important role of various sulphur compounds in model systems of boiled beef [47]. In sheep meat, 4,6-dimethyl-1,3-oxathiane in particular, has been found in high concentrations in the fat of non-castrated males (rams) and has been described as having a barn/animal aroma [88].

The sulphur in those compounds is derived from amino acids (glutathione, methionine, cysteine, and cystine) and thiamine, and it is liberated when meat is cooked, although it can be released earlier, during fermentation in the rumen [39]. It is generally accepted that in the early stages of cooking, glutathione is the principal source of H_2_S, but cysteine fulfills this fundamental role in the latter stages of cooking [46].

## 7. Conclusions

Lipid oxidation, Maillard and Strecker reactions are the main reactions potentially responsible for the odorous compounds known to occur in cooked meat, whereas the degradation of thiamin seems to play a minor role. The same reaction, such as lipid oxidation, can have different effects on the aroma, depending on the degree and the time that occurs (in raw, cooked, or reheated meat). Those chemical reactions are the same for any type of meat, and quantitative more than qualitative differences in the volatile profile might explain the distinct aroma between species. In the case of ruminant meat, several aroma compounds or precursors can be formed in the rumen, such as 12-trimethyl-decanal, indoles or branched chain fatty acids, but their actual role in the overall cooked meat aroma is still controversial.

Knowing the source of aromatic compounds is essential to understanding, controlling, and improving the quality of meat products. Numerous compounds have been proposed (sulphur compounds, pyrazines, aldehydes, ketones, phenols, organic acids, among others) as responsible for the aroma of the meat. As in the case of 2-methyl-3-furanthiol, the same aroma can arise through different pathways of formation.

Nowadays, new odour active compounds are still being discovered, and further studies are necessary to quantify them. More effort should be also focus in reconstitution experiments, taken into account the food matrix, to fully understand the role of each aroma compound identified in the cooked meat and their possible effect in consumer acceptability.
